# Supplementary material for: Technological impact, generality, and complementarity of artificial intelligence patents: Evidence from Samsung Electronics
Source: PLoS One. 2026 May 5;21(5):e0346056. doi: 10.1371/journal.pone.0346056 (PMC13143100; doi:10.1371/journal.pone.0346056)
Supplement: S1 Dataset — (DOCX) [file pone.0346056.s001.docx]

**Technological Impact, Generality, and Complementarity of Artificial Intelligence Patents: Evidence from Samsung Electronics**

**SUPPORTING INFORMATION**

Sangrok Lee1, Taehyun Jung*1**

*1Graduate School of Technology & Innovation Management, Hanyang University, Seoul, Republic of Korea*

* Corresponding author E-mail: tjung@hanyang.ac.kr

**S1 Dataset. Patent dataset underlying the findings of this study.** This dataset contains all granted patents filed by Samsung Electronics between 1982 and 2018, including forward citation counts, generality index scores, technological complementarity measures, AI classification indicators, coarsened exact matching variables, and associated control variables used in the empirical analyses. The file is provided in csv format at https://sites.google.com/view/taehyunjung/research/ai-patent.

**APPENDIX I**

**A detailed procedure for identifying the patens assigned to Samsung Electronics**

Specifically, we:

1. Established clear inclusion criteria: patents assigned to Samsung Electronics and its wholly-owned subsidiaries sharing integrated R&D systems (Samsung Austin Semiconductor, Samsung Guangzhou Electronics, and other verified overseas subsidiaries) and Samsung Display (including Samsung Mobile Display) which was spun off in 2012 from Samsung Electronics but is still 84.8% owned affiliate.
2. Excluded patents from legally distinct Samsung affiliates with separate management and R&D operations such as Samsung Electro-Mechanics (23.7% owned), Samsung SDS (23% owned), Samsung SDI (19.58% owned) etc., and unrelated entities with similar names.
3. Cross-referenced each USPTO patent number with PATSTAT’s psn_name field to verify assignee identity, under the same inclusion criteria, manually reviewing ambiguous cases.

**Table A1. Definitions of 8 Categories of AIPD (Giczy et. al., 2022)**

| Categories of AIPD | Definition |
| --- | --- |
| Knowledge Processing (KR) | Methods to represent facts about the world and to derive new facts (or knowledge) from a knowledge base. For example, expert systems. |
| Speech | Speech recognition includes methods to understand a sequence of words given an acoustic signal. For example, the noisy channel model. |
| AI Hardware | The field of AI hardware includes physical hardware designed to implement artificial intelligence software. AI hardware may include logic circuitry, memory, video, processors, and solid-state technologies. It may also include embedded software. |
| Natural Language Processing (NLP) | Methods for understanding and using data encoded in human natural language. For example, language models. |
| Machine Learning (ML) | A broad class of computational learning models. For example, supervised learning classification models are algorithms that learn to classify observations based on pre-labeled training data. |
| Computer vision | Methods to extract and understand information from visual input, including images and videos. |
| Planning/Control | Methods to identify and execute plans to achieve specified goals. |
| Evolutionary computation (EVO) | Contains a set of computational methods utilizing aspects of nature and, specifically, evolution. For example, genetic algorithms |

**APPENDIX II**

**Comparison of JTH Generality and Shannon Generality**

As illustrated in Figure A1, Panel (a), when 10 citing patents are uniformly distributed across 10 distinct CPC classes, the Shannon generality reaches approximately 3.32, while the corresponding JTH generality is 0.9. When 200 citing patents are evenly distributed across 200 classes, Shannon generality increases to 7.64, whereas JTH generality only slightly increases to 0.995. Conversely, in a highly uneven case—e.g., where 9 out of 10 citing patents fall into one CPC class and only one cites from another—Shannon generality remains higher and increases more steeply than JTH generality, as shown in Panel (b). These results indicate that JTH generality tends to compress variance in high-diversity scenarios, whereas Shannon generality offers greater sensitivity to variations in citation dispersion.


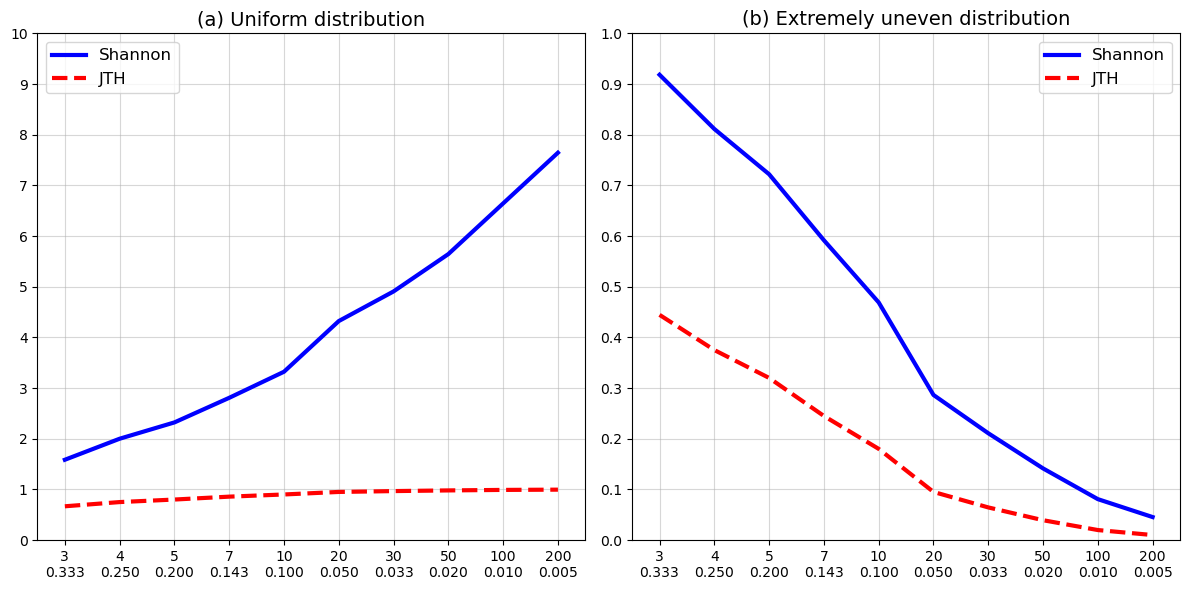


Figure A1 Simulation results of two generality measures

Note: Numbers on the horizontal axis represent the number of citing patents (𝑛) and the probability of one CPC class (𝑆). In Panel (a), the probability is evenly distributed; in Panel (b), all but one citing patent are concentrated in a single CPC class.

**APPENDIX III**

**Table A2 Average Marginal Effects (AME) and CI on Impact and Generality**

|  | **Model A2-1** | **Model A2-2** | **Model A2-3** | **Model A2-4** | **Model A2-5** |
| --- | --- | --- | --- | --- | --- |
|  | FC(5yr)  ZINB | JTH(5yr)  Tobit | Shannon(5yr)  Tobit | JTH(5yr)  Heckman | Shannon(5yr)   Heckman |
| AI Patent | 0.342^**^ | 0.085^***^ | 0.209^***^ | 0.020^**^ | 0.052^**^ |
|  | [0.081,0.603] | [0.030,0.140] | [0.082,0.336] | [0.001,0.039] | [0.007,0.097] |
|  |  |  |  |  |  |
| Number of Claims | 0.042^***^ | 0.012^***^ | 0.029^***^ | 0.001^*^ | 0.003^**^ |
|  | [0.032,0.053] | [0.008,0.017] | [0.019,0.040] | [-0.000,0.003] | [0.000,0.006] |
|  |  |  |  |  |  |
| Number of Inventors | 0.132^***^ | 0.024^***^ | 0.058^***^ | -0.007^**^ | -0.014^**^ |
|  | [0.060,0.205] | [0.014,0.034] | [0.034,0.083] | [-0.012,-0.001] | [-0.027,-0.001] |
|  |  |  |  |  |  |
| ln(Number of CPC Codes) | 0.461^***^ | 0.122^***^ | 0.287^***^ | 0.006 | 0.012 |
|  | [0.375,0.546] | [0.089,0.155] | [0.209,0.364] | [-0.008,0.021] | [-0.022,0.046] |
|  |  |  |  |  |  |
| ln(Backward Citations) | 0.132^***^ | 0.049^***^ | 0.114^***^ | 0.011^**^ | 0.026^**^ |
|  | [0.045,0.220] | [0.016,0.081] | [0.037,0.191] | [0.001,0.021] | [0.003,0.049] |
|  |  |  |  |  |  |
| ln(Other References) | -0.059 | -0.001 | -0.001 |  |  |
|  | [-0.147,0.030] | [-0.029,0.027] | [-0.065,0.063] |  |  |
|  |  |  |  |  |  |
| Days to Grant | -0.002^***^ | -0.000^***^ | -0.001^***^ |  |  |
|  | [-0.002,-0.002] | [-0.000,-0.000] | [-0.001,-0.001] |  |  |
|  |  |  |  |  |  |
| ln(Family Size) | 0.272^***^ | 0.001 | 0.008 | -0.033^***^ | -0.069^***^ |
|  | [0.158,0.385] | [-0.033,0.034] | [-0.071,0.087] | [-0.046,-0.020] | [-0.099,-0.039] |
|  |  |  |  |  |  |
| ln(Forward Citations) |  |  |  | 0.118^***^ | 0.403^***^ |
|  |  |  |  | [0.103,0.133] | [0.368,0.438] |
| Observations | 10695 | 10695 | 10695 | 10695 | 10695 |

Notes: Average marginal effects (AME) with 95% confidence intervals in brackets.

Model R2-1: Zero-inflated negative binomial for 5-year forward citations.

Models R2-2 & R2-3 Tobit models for JTH and Shannon generality without FC controls.

Models R2-4 & R2-5: Heckman selection models for JTH and Shannon generality.

All models (except Heckman) include filing year and WIPO major field fixed effects.

Heckman models exclude fixed effects due to convergence issues. Standard errors clustered by filing year. * p<0.10, ** p<0.05, *** p<0.01

**APPENDIX IV**

**Table A3. Adjusted forward citation count (Percentile and z-score)**

|  | **Model A3-1**  (Tobit) | **Model A3-2**  (Tobit) | **Model A3-3**  (OLS) | **Model A3-4**  OLS |
| --- | --- | --- | --- | --- |
|  | 5-yr FW citations   percentile | 5-yr FW percentile,  w/o self-citations | 5-yr FW citations  Z-score | 5-yr FW Z-score,  w/o self-citations |
| AI patent | 1.768*** | 1.790*** | 0.063* | 0.073** |
|  | (0.515) | (0.504) | (0.027) | (0.024) |
| Number of claims | 0.358*** | 0.357*** | 0.011*** | 0.012*** |
|  | (0.044) | (0.041) | (0.001) | (0.001) |
| Number of inventors | 0.822*** | 0.702*** | 0.031** | 0.026* |
|  | (0.092) | (0.111) | (0.010) | (0.010) |
| Number of CPCs (ln) | 3.336*** | 3.221*** | 0.107*** | 0.104*** |
|  | (0.297) | (0.332) | (0.015) | (0.016) |
| Backward citations (ln) | 0.971*** | 0.811*** | 0.026* | 0.020 |
|  | (0.210) | (0.234) | (0.011) | (0.011) |
| Other references (ln) | -0.226 | -0.126 | -0.008 | -0.006 |
|  | (0.399) | (0.329) | (0.012) | (0.010) |
| Days to grant | -0.015*** | -0.014*** | -0.000*** | -0.000*** |
|  | (0.001) | (0.001) | (0.000) | (0.000) |
| Patent family size (ln) | 1.945*** | 1.366* | 0.080*** | 0.057** |
|  | (0.556) | (0.568) | (0.016) | (0.017) |
| Constant | 53.851*** | 53.574*** | 0.067 | 0.064 |
|  | (3.845) | (3.846) | (0.098) | (0.099) |
| Observations | 10,695 | 10,695 | 10,695 | 10,695 |
| R^2^ |  |  | 0.053 | 0.044 |
| Log likelihood | -49350 | -49234 |  |  |
| Notes:  Robust standard errors clustered by filing year in parentheses. *** p<0.001, ** p<0.01, * p<0.05  All models include filing year and technology field (WIPO) fixed effects.  The dependent variable in Models 5-6 is the count of forward citations transformed as percentile and rank variables regarding temporal trends.  Models 5-6 use Tobit regression with citations percentile (censored at 1 and 100) as the dependent variable.  Models 7-8 use OLS with standardized (z-score) citations as the dependent variable. | | | | |

**APPENDIX V**

**Table A4. Sensitivity analysis using 3-year time windows for technological impact**

|  | **Model A4-1**  (NBREG) | **Model A4-2**  (Tobit) | **Model A4-3**  (OLS) |  |
| --- | --- | --- | --- | --- |
|  | 3-yr FW   citations | 3-yr FW percentile,  w/o self-citations | 3-yr FW Z-score,  w/o self-citations |  |
| AI patent | 0.174* | 1.099* | 0.038 |  |
|  | (0.082) | (0.429) | (0.025) |  |
| Number of claims | 0.023*** | 0.267*** | 0.010*** |  |
|  | (0.003) | (0.045) | (0.001) |  |
| Number of inventors | 0.078*** | 0.703*** | 0.030* |  |
|  | (0.017) | (0.109) | (0.011) |  |
| Number of CPCs (ln) | 0.242*** | 2.365*** | 0.096*** |  |
|  | (0.028) | (0.364) | (0.017) |  |
| Backward citations (ln) | 0.092** | 0.786** | 0.031* |  |
|  | (0.029) | (0.251) | (0.012) |  |
| Other references (ln) | -0.007 | 0.166 | 0.005 |  |
|  | (0.027) | (0.259) | (0.012) |  |
| Days to grant | -0.001*** | -0.014*** | -0.000*** |  |
|  | (0.000) | (0.001) | (0.000) |  |
| Patent family size (ln) | 0.126** | 1.450*** | 0.069*** |  |
|  | (0.042) | (0.428) | (0.017) |  |
| Constant | -0.373 | 54.051*** | 0.072 |  |
|  | (0.251) | (3.853) | (0.097) |  |
| lnalpha | 0.778*** |  |  |  |
|  | (0.095) |  |  |  |
| Observations | 10,695 | 10,695 | 10,691 |  |
| (Pseudo) R^2^ | 0.0826 |  | 0.064 |  |
| Log likelihood | -11310 | -48223 |  |  |
| Notes:  Robust standard errors clustered by filing year in parentheses. *** p<0.001, ** p<0.01, * p<0.05  All models include filing year and technology field (WIPO) fixed effects.  Model 9 uses negative binomial regression.  Model 10 uses Tobit regression with percentile DV (censored at 1-100).  Model 11 uses OLS with standardized (z-score) DV. | | | | |

**APPENDIX VI**

**Computational Procedures for Technological Complementarity Measurement**

This appendix provides detailed documentation of the computational procedures used to calculate the technological complementarity scores reported in the main analysis.

Our complementarity measure builds on Teece et al.’s (1994) conceptualization of technological relatedness by quantifying the extent to which technology combinations within patents occur more frequently than expected by chance. We operationalize this through a normalized co-occurrence index that compares observed technology pairings against a null model of random technology assignment, using the hypergeometric distribution to account for sampling without replacement.

**Step-by-Step Procedure**

1. **Step 1: Construct dynamic knowledge matrix**

For each Samsung Electronics patent filed in year *t* (where *t* ranges from 1982 to 2018), we construct a knowledge matrix based on all USPTO patents filed during the 5-year window [*t*-5, *t*-1]. This rolling window approach captures the evolving technological landscape while maintaining sufficient sample size for reliable statistical estimation. For patents filed in years 1982-1986, we use all available prior patents as the reference population.

1. **Step 2: Extract technology classifications**

Within each 5-year window, we extract all CPC codes at the 4-digit subclass level (e.g., H04N, G06F) assigned to patents. For each window *w*, we calculate:

$N_{j}^{w}$= total count of patents containing technology *j*

where *j* indexes all distinct CPC subclasses present in window *w*. This provides the marginal distribution of each technology’s prevalence.

1. **Step 3: Compute observed co-occurrence matrix**

For each window *w*, we construct the observed co-occurrence matrix $C^{w}$ by counting how many patents contain both technologies *j* and *k* simultaneously:

$$C_{\mathrm{jk}}^{w}=\left| \left\{ p\in w:j\in p\wedge k\in p \right\} \right|$$

This creates a symmetric matrix where diagonal elements are undefined (a technology cannot co-occur with itself). The matrix dimensions vary across windows depending on the number of distinct technologies present.

1. **Step 4: Calculate expected co-occurrence under null model**

Under the null hypothesis of random technology assignment (i.e., no systematic complementarity or substitution), the expected co-occurrence follows the hypergeometric distribution. For each technology pair (*j*, *k*) in window *w*, we calculate:

$$\mu_{\mathrm{jk}}^{w}=\frac{N_{j}^{w}\times N_{k}^{w}}{N^{w}}$$

where $N^{w}$represents the total number of patents in window *w*. This expected value reflects how frequently technologies *j* and *k* would co-occur if their assignments were independent.

1. **Step 5: Compute standard deviation**

The variance of co-occurrence under the hypergeometric distribution accounts for sampling without replacement. We calculate the standard deviation as:

$$\sigma_{\mathrm{jk}}^{w}=\sqrt{\mu_{\mathrm{jk}}^{w}\times\left( 1-\frac{N_{j}^{w}}{N^{w}} \right)\times\left( 1-\frac{N_{k}^{w}}{N^{w}} \right)\times\left( \frac{N^{w}-N_{j}^{w}-N_{k}^{w}}{N^{w}-1} \right)}$$

This formula adjusts for the finite population size and accounts for the constraint that each patent can contain each technology at most once.

1. **Step 6: Calculate raw complementarity index**

For each technology pair (*j*, *k*) in window *w*, we compute the standardized co-occurrence deviation:

$$\lambda_{\mathrm{jk}}^{w}=\frac{C_{\mathrm{jk}}^{w}-\mu_{\mathrm{jk}}^{w}}{\sigma_{\mathrm{jk}}^{w}}$$

This raw complementarity index represents how many standard deviations the observed co-occurrence deviates from the null expectation. Positive values indicate technologies that co-occur more frequently than chance (complementarity); negative values indicate technologies that co-occur less frequently than chance (substitution).

1. **Step 7: Normalize to [0,1] Interval**

To facilitate interpretation and ensure comparability across windows with different technology distributions, we apply min-max normalization within each window:

$$\lambda_{\mathrm{jk}}^{'w}=\frac{\lambda_{\mathrm{jk}}^{w}-\min\left( \lambda^{w} \right)}{\max\left( \lambda^{w} \right)-\min\left( \lambda^{w} \right)}$$

This transformation maps all complementarity values to [0,1], where:

- $\lambda’_{jk}^{w}$ > 0.5 indicates complementary relationships (co-occurrence exceeds expectation)
- $\lambda’_{jk}^{w}$ < 0.5 indicates substitutive relationships (co-occurrence falls below expectation)
- $\lambda’_{jk}^{w}$ = 0.5 indicates independence (co-occurrence matches expectation)

1. **Step 8: Extract pairwise complementarities for focal patents**

For each focal Samsung patent *i* filed in year *t* containing $n_{i}$ CPC subclasses {$j_{1}$, $j_{2}$*, ...,*$j_{n-1}$}, we:

- Identify all unique technology pairs: (*j_a_, j_b_*) where *a* < *b*, yielding $m_{i}$= *n_i_*(*n_i_*_-1_)/2 pairs
- Retrieve the corresponding $\lambda’_{jk}$ values from the knowledge matrix for window [*t*-5, *t*-1]
- If any technology pair is absent from the reference window (occurs when a technology is newly introduced), we exclude that pair from the calculation

1. **Step 9: Calculate Patent-Level Complementarity Score**

For each patent *i*, we compute the complementarity score as the arithmetic mean of all pairwise normalized complementarity indices:

$$\mathrm{Complementarity}\mathrm{Score}_{i}= \left( \frac{1}{m_{i}} \right)\times\sum_{a<b} \lambda_{j_{a}j_{b}}^{'}$$

where the sum ranges over all technology pairs within patent *i* that have computable complementarity values from the reference window. Patents containing only a single CPC subclass ($n_{i}$= 1) have undefined complementarity scores and are excluded from complementarity analyses.
