# Supplementary material for: Technological impact, generality, and complementarity of artificial intelligence patents: Evidence from Samsung Electronics
Source: PLoS One. 2026 May 5;21(5):e0346056. doi: 10.1371/journal.pone.0346056 (PMC13143100; doi:10.1371/journal.pone.0346056)
Supplement: Attachment — Submitted filename: Response PLOSONE- 08.09.2025.docx [file pone.0346056.s003.docx]

The paper entitled „Artificial Intelligence as a General-Purpose Technology? Insights from Samsung’s Patents” has an interesting topic based on using AI and modelling. But in order to be proposed for publication, the authors need to make the following improvements:

1.**The Introduction** is well done, an dis following the rules of writing it.

2.The **THEORETICAL BACKDROP & HYPOTHESIS DEVELOPMENT** is suffering of adding new and updated sources. As the authors use a topic and analyse it under the impact of AI, the authors need to use upadated references. They need to improve this weaknesses as follows: at every sub-section from the second section, the authors need to add new and updated sources especially from 2023-2025 (they used sources from 1990, 2001, 2010, 2020). So is very important, not only for the journal, but also for the readers.

3.**The Methodology** is well done.

4.**Contribution to Literature** must be improved. The authors used only one source (from 1995), so to measure the gap between the study and the literature in the filed, they have to use a few new and updated sources.

5.**Practical implications** need also a more carefull description. It is indicated to describe PI on categories (write them using italic writing for sub-titles): for employees, for managers, for the industry, for society. Being such a challenging and dynamic field, it is requiered such a description based on results and innovation.

6.References must be updated, as the requirement, being a dynamic field under the impact of AI.

Having these proposals in view, the paper, receives minor revision.
