# Supplementary material for: Technological impact, generality, and complementarity of artificial intelligence patents: Evidence from Samsung Electronics
Source: PLoS One. 2026 May 5;21(5):e0346056. doi: 10.1371/journal.pone.0346056 (PMC13143100; doi:10.1371/journal.pone.0346056)
Supplement: Attachment — Submitted filename: Response to editor and reviewers_PONE-D-25-42932.docx [file pone.0346056.s004.docx]

**AUTHOR’S RESPONSE TO EDITOR AND REVIEWERS**

**Manuscript ID:** PONE-D-25-42932

**Title:** Artificial Intelligence as a General-Purpose Technology? Insights from Samsung’s Patents

Dear Editor and Reviewers,

We sincerely thank you for your thoughtful and constructive feedback on our manuscript. Your comments have significantly improved the theoretical clarity, empirical rigor, and overall presentation of our work.

In response, we have undertaken major revisions across multiple dimensions of the paper—including theoretical reframing, methodological refinements, additional robustness checks, and expanded discussion and conclusion sections. We have also thoroughly reviewed our data sample, cross-verified name matching using a third-party database, and updated the analytical dataset accordingly.

Below, we provide a point-by-point response to each comment, detailing the revisions made.

Sincerely,

Taehyun Jung

(on behalf of all authors)

**AUTHOR’S RESPONSE TO EDITOR’S COMMENTS**

**Editor Comment 1:**

*One of my main concerns is that I am not fully convinced that the framing of general-purpose technologies match your empirical analysis. The fact that AI-patents received more citations and are more widely applicable does not necessarily make them general purpose technologies. More clarification on this essential aspect is required.*

**Author’s Response:**

We appreciate this critical observation. In response, we have substantially revised our theoretical framework to more fully address the concern. Most notably, we have introduced a third hypothesis on technological complementarity to reflect the notion of innovation complementarities drawn from the GPT literature (Bresnahan & Trajtenberg, 1995). With this addition, the revised manuscript now addresses all three defining characteristics of general-purpose technologies—technological impact, generality, and complementarity.

To reflect these changes, we have also removed the term “GPT” from the title and instead phrased it in terms of the specific technological characteristics examined throughout the manuscript. This decision improves clarity and aligns the title more closely with the empirical focus of the study.

Finally, in the revised Discussion and Conclusion sections, we provide a more explicit reflection on the limitations of firm-level patent data in definitively establishing GPT status. We clarify that, while our findings are consistent with key GPT attributes, additional macroeconomic and multi-firm analyses would be needed to make broader claims about AI’s general-purpose nature.

**Editor Comment 2:**

*I agree with Reviewer 1 that your theory section requires a revision. However, my main concern is not the age of the references but rather the disintegration of different arguments. It is, for instance, not fully clear whether and how absorptive capacity matters for your study. In this context, it is also required to embed the development of hypotheses much better in the literature (instead of having them as stand-alone items). More work needs to be done to connect your literature review to the hypotheses.*

**Author’s Response:**

We appreciate the editor’s observation and agree that the previous version of the theory section lacked integration across its core arguments. In response, we have substantially restructured the theoretical framework to enhance logical flow, eliminate tangential elements, and more clearly embed the development of our hypotheses within the relevant literature.

Key changes include:

1. Streamlined theoretical narrative:

We removed the discussion of absorptive capacity, as it was not essential to our central arguments concerning AI’s technological characteristics. The revised theory section now focuses on three tightly integrated components:

(a) the foundational literature on general-purpose technologies (GPTs) and their relevance to AI;

(b) the conceptual mapping of GPT attributes (impact, generality, complementarity) to observable patent-based indicators; and

(c) the firm-level innovation context through which these attributes are examined.

2. Improved hypothesis integration:

Each hypothesis is now directly and explicitly connected to the preceding theoretical discussion. Rather than appearing as stand-alone statements, the hypotheses emerge naturally from the conceptual arguments and are anchored in specific GPT dimensions. This restructuring improves both conceptual coherence and alignment with prior literature.

We believe these revisions have significantly strengthened the theoretical foundation of the study and addressed the editor’s concern regarding integration between literature and hypothesis development.

**Editor Comment 3:**

*The concluding and discussion section is underdeveloped and needs thorough revision and a more in-depth discussion of the implications of your work.*

**Author’s Response:**

We appreciate the editor’s feedback regarding the need for a more developed concluding section. In response, we have completely rewritten and substantially expanded the Discussion and Conclusion sections to provide a more in-depth and integrated treatment of the implications of our findings.

The revised sections now include a thorough discussion of the theoretical contributions, practical implications, boundary conditions and limitations, and future research directions. Specifically, we articulate three core contributions of this study (see pp. 52–53, Theoretical Contributions subsection):

1. Firm-level empirical evidence for GPT-like characteristics of AI technologies, addressing an important gap in the existing literature;
2. Methodological innovation through the integration of multiple patent-based indicators (impact, generality, complementarity) within a longitudinal firm-level analysis; and
3. Temporal documentation of the evolution of AI-related innovation patterns within a major global technology firm.

We also provide specific and actionable implications based on our empirical results. For practitioners, we discuss how AI category heterogeneity can inform R&D portfolio diversification and strategic resource allocation. For policymakers, we connect the findings on generality and complementarity to broader technology infrastructure and innovation policy—highlighting the importance of supporting ecosystems that facilitate AI’s integration with other technological domains.

We believe these revisions substantially strengthen the manuscript and address the editor’s request for a more comprehensive and meaningful concluding discussion.

**Editor Comment 4:**

*I request to strengthen the argument about why you look at the patents of a single company. Most arguments that you bring up could be easily addressed in an econometric study with company dummies or fixed effects.*

**Author’s Response:**

Thank you for this important comment. We agree that some forms of firm-level heterogeneity can be addressed through econometric techniques such as firm dummies or fixed effects. However, our dependent variables are not limited to raw citation counts; rather, they include measures of citation breadth (technological, geographical and organizational) and technological complementarity—which depend on the contextual and structural characteristics of the citing patents (as shown by Jaffe, 1993 and the following studies).

These characteristics are known to be influenced by organizational and geographic proximity as well as by firm-specific technological trajectories. Accurately modeling these layered interactions would require a complex research design involving numerous control variables or multi-level fixed effects, including but not limited to: firm identity, firm location, technology domain, and network-based relationships among patents. Given the exploratory and theory-building nature of this study, we opted for a single-firm design to reduce model complexity and eliminate unobserved heterogeneity from organizational or strategic differences across firms—while acknowledging a tradeoff in external generalizability.

We have now incorporated this rationale into the Methodology section and more clearly discussed the strengths, limitations, and generalizability of our single-firm research design in the revised manuscript.

**Editor Comment 5:**

*Given the results in Table 5, I am really wondering what is driving the results for the generality indices in Table 4.*

**Author’s Response:**

Thank you for this insightful question regarding the heterogeneous effects of AI subcategories on the overall generality findings. We appreciate the opportunity to clarify how different AI domains contribute to the aggregate results shown in Table 5-a.

Our analysis reveals that the high generality of AI patents is primarily driven by specific AI domains rather than being uniformly distributed across all AI categories. As shown in Table 10 (Model 45), when we decompose AI patents into eight subcategories with Non-AI patents as the reference category:

- Vision (0.053***), Speech (0.071*), and Machine Learning (0.155*) patents demonstrate significantly higher generality compared to Non-AI patents
- In contrast, Evolutionary computing actually exhibits lower generality than Non-AI patents (-0.138*)
- Other domains such as Hardware, Knowledge processing, NLP, and Planning show no significant differences from Non-AI patents in terms of generality

Table 11 further illuminates the within-AI heterogeneity by comparing AI domains directly against each other, using Vision as the baseline. The results (Model 47) confirm that:

- Vision patents exhibit the highest generality among AI domains
- Machine Learning shows comparable generality to Vision (coefficient: 0.092, not significant)
- Speech also demonstrates similar generality levels to Vision
- Most other AI domains (Evolutionary computing, Hardware, Knowledge processing, Planning) have significantly lower generality than Vision

These findings suggest that the aggregate positive effect of AI on technological generality reported in Table 5-a is concentrated in application-oriented AI domains (Vision, Speech, and Machine Learning) that tend to span multiple technological fields. In contrast, more specialized or infrastructure-oriented AI domains show limited or even negative effects on generality. This heterogeneity provides important nuance to our main findings and demonstrates that not all AI technologies exhibit general-purpose characteristics equally.

**Editor Comment 7:**

*You need to define what an AI patent is early on. Are the AI patents on page 10 also identified using that database that is mentioned later on?*

**Author’s Response:**

Thank you for this comment. In response, we have revised the Introduction to define what constitutes an AI patent early in the manuscript. Specifically, we now clarify that:

“To identify AI-related patents, we use the USPTO Artificial Intelligence Patent Dataset (AIPD), which employs machine learning techniques to classify patents into eight distinct AI technology categories (Giczy et al., 2022).”

We confirm that the AI patents referenced on page 10 of the original manuscript and section 3.5 of the revision—and throughout the empirical analysis—are identified using this same dataset.

**Editor Comment 8:**

*What is the number of observations in the regression tables, the descriptive statistics, and the correlations? In addition to addressing these comments, I expect you to address the Reviewer comments very carefully. Please do also take care that all references are shown in the revised version and that the correlation table contains the variable names.*

**Author’s Response:**

Thank you for your detailed suggestions for improvement. We have carefully addressed all presentation issues.

**Changes made:**

1. **Added observation counts:** All regression tables now clearly report the number of observations. For our main analyses using the CEM-matched sample: N = 10,695 (see Tables 4, 5-a, 5-b, and 6, bottom rows).
2. **Fixed correlation table:** Table 2 now includes proper variable names in the first column matching the descriptive statistics labels (see page 20, Table 2).
3. **Verified all references:** We have checked that all in-text citations appear in the reference list and vice versa. Several references that were incorrectly formatted or missing have been added (see pages 60-67, References section).
4. **Fixed cross-reference errors:** All “Error! Reference source not found” placeholders have been corrected with verifying all references (see throughout manuscript).
5. **Added sample sizes to descriptive tables:** Table 1 now reports the full sample size (N = 86,824 patents) and the matched sample size (N = 10,695) with clear labels (see page 19, Table 1 note).

**AUTHOR’S RESPONSE TO REVIEWER 1**

We thank Reviewer 1 for the constructive feedback and appreciation of our topic and methodology.

**Reviewer 1, Comment 1:**

*The Introduction is well done, and is following the rules of writing it.*

**Author’s Response:**

Thank you for your encouraging feedback.

**Reviewer 1, Comment 2:**

*The THEORETICAL BACKDROP & HYPOTHESIS DEVELOPMENT is suffering of adding new and updated sources. As the authors use a topic and analyze it under the impact of AI, the authors need to use updated references. They need to improve this weakness as follows: at every sub-section from the second section, the authors need to add new and updated sources especially from 2023-2025 (they used sources from 1990, 2001, 2010, 2020).*

**Author’s Response:**

Thank you for this helpful suggestion. We agree that incorporating recent literature is particularly important in a rapidly evolving field such as AI. In response, we have substantially updated the theoretical section by integrating more recent studies (2022–2025) while also retaining key foundational references that remain critical for grounding our conceptual framework.

We believe that the revised literature review and hypothesis development now strike a better balance between classic theoretical insights and the most current empirical and conceptual contributions.

We added nine recent references (2022–2025) across the revised manuscript:

- Hötte et al. (2022), Lee et al. (2025); for hypotheses: Qin et al. (2025), Rammer et al. (2022), Lee et al. (2022), Czarnitzki et al. (2023), Jiang et al. (2022 ), Hoffreumon et al. (2024), Jiang et al. (2022)

**Reviewer 1, Comment 3:**

*The Methodology is well done.*

**Author’s Response:**

We appreciate this positive assessment.

**Reviewer 1, Comment 4:**

*Contribution to Literature must be improved. The authors used only one source (from 1995), so to measure the gap between the study and the literature in the field, they have to use a few new and updated sources.*

**Author’s Response:**

Thank you for your constructive suggestion. In response, we have completely rewritten and substantially expanded the "Contribution to Literature" section to better articulate our study’s positioning within recent scholarly debates and to reflect the rapid developments in AI, GPT, and innovation research.

The revised section now outlines three distinct contributions, each supported by updated references from 2022–2025:

1. Theoretical contribution to GPT research: We contextualize our findings within the recent literature on general-purpose technologies, highlighting how our firm-level, patent-based analysis complements macro-level or conceptual studies of AI as a GPT (e.g., Bresnahan, 2024; Hötte et al., 2022).
2. Methodological contribution to innovation measurement: We demonstrate the value of combining multiple patent-based indicators (impact, generality, complementarity) and draw on recent methodological advancements in innovation studies and patent analytics (e.g., Qin et al., 2025; Czarnitzki et al., 2023).
3. Empirical contribution to AI innovation literature: We explicitly compare our study to recent empirical analyses of AI patents and innovation dynamics (e.g., Rammer et al., 2022; Jiang et al., 2022; Hoffreumon et al., 2024), and we highlight how our work extends this literature by examining AI’s performance relative to non-AI technologies within a single-firm longitudinal context.

In addition, we now clearly position our contribution within ongoing scholarly debates on 1)AI’s transformative potential and GPT status; 2) the challenges of measuring innovation in fast-evolving technological domains; and 3) the value of firm-level perspectives in complementing macroeconomic or industry-level studies

We believe these revisions significantly strengthen the contribution of our manuscript and better demonstrate how it advances current knowledge in the field.

**Reviewer 1, Comment 5:**

*Practical implications need also a more careful description. It is indicated to describe PI on categories (write them using italic writing for sub-titles): for employees, for managers, for the industry, for society. Being such a challenging and dynamic field, it is required such a description based on results and innovation.*

**Author’s Response:**

Thank you for this valuable suggestion. In response, we have substantially expanded and reorganized the Practical Implications section to provide a more structured and targeted discussion. The revised section now follows the requested format with clearly labeled subheadings, and each implication is explicitly grounded in our empirical findings.

**Changes made:**

1. Structured implications across key stakeholder groups:
   A. For Employees: While this category yielded limited direct insights in our study, we briefly discuss the implications of AI-induced innovation shifts for future skill demands and employee retraining, particularly in domains with high generality such as machine learning.
   B. For Managers: We outline strategic recommendations regarding innovation portfolio diversification, R&D resource allocation, and capability-building. These are informed by our findings on AI’s impact, generality, and complementarity, as well as by heterogeneity across AI categories.
   C. For Industry: We discuss broader implications for technology adoption, standard-setting, and cross-sector collaboration. In particular, we highlight how AI's complementarity suggests the need for interoperability strategies and alliance formation in key subfields (e.g., Vision and Speech AI).
   D. For Society and Policymakers: We connect our results to policy considerations, emphasizing the need for infrastructure-oriented investment and targeted support. Our findings on generality and complementarity support treating AI as a foundational technology that warrants public investment—particularly in enabling technologies and data ecosystems.
2. Empirical grounding:

Each implication is directly linked to one or more of our core findings—technological impact, generality, or complementarity—to ensure that the recommendations are evidence-based rather than generic.

3. Attention to AI category heterogeneity:

Drawing on our heterogeneity analysis, we offer nuanced strategic and policy implications. For instance, we discuss how Vision AI’s high complementarity may call for co-investment strategies, while Machine Learning’s high generality may be better suited for platform-based deployment models.

We believe these revisions significantly strengthen the practical relevance of our study and offer more actionable insights for different stakeholders engaged in navigating the dynamic landscape of AI-driven innovation.

**Reviewer 1, Comment 6:**

*References must be updated, as the requirement, being a dynamic field under the impact of AI.*

**Author’s Response:**

Thank you for pointing this out. As noted in Author’s Response to Comment 2, we have added 9+ recent references from 2022-2025 throughout the manuscript, particularly in the theoretical sections and discussion. The reference list now includes contemporary work on AI innovation, recent patent studies, and updated research on GPTs and transformative technologies (see pages 60-67, References section).

**Changes made (Updated references):**

Bresnahan, T. (2024). What innovation paths for AI to become a gpt? Journal of Economics & Management Strategy, 33(2), 305-316. https://doi.org/https://doi.org/10.1111/jems.12524

Dibiaggio, L., Nasiriyar, M., & Nesta, L. (2014). Substitutability and complementarity of technological knowledge and the inventive performance of semiconductor companies. Research Policy, 43(9), 1582-1593.

Hoffreumon, C., Forman, C., & Van Zeebroeck, N. (2024). Make or buy your artificial intelligence? Complementarities in technology sourcing. Journal of Economics & Management Strategy, 33(2), 452-479.

Hötte, K., Tarannum, T., Verendel, V., & Bennett, L. (2022). Exploring Artificial intelligence as a general purpose technology with patent data--a systematic comparison of four classification approaches. arXiv e-prints, arXiv-2204.

Jiang, L., Chen, J., Bao, Y., & Zou, F. (2022). Exploring the patterns of international technology diffusion in AI from the perspective of patent citations. Scientometrics, 127(9), 5307-5323.

Lee, S., Hwang, J., & Cho, E. (2022). Comparing technology convergence of artificial intelligence on the industrial sectors: two-way approaches on network analysis and clustering analysis. Scientometrics, 127(1), 407-452.

Lee, S., Hwang, J., & Cho, E. (2025). Dynamic patterns of AI technology diffusion: focusing on time series clustering and patent analysis. Scientometrics, 130(4), 2005-2036.

Qin, L., Xie, W., & Chen, X. (2025). The impact of knowledge coupling on technological generality in AI: The moderating role of collaborative innovation network centralities. IEEE Transactions on Engineering Management.

**AUTHOR’S RESPONSE TO REVIEWER 2**

We are grateful for Reviewer 2’s thorough and constructive review. The detailed methodological suggestions have substantially strengthened our empirical approach.

**Reviewer 2, Overall Assessment Recognition:**

**Author’s Response:**

We appreciate Reviewer 2’s recognition that “The study is promising and mostly well designed” and that “The within-firm design is a clear strength.” We have carefully addressed all methodological concerns raised.

**Major Comments**

**Reviewer 2, Major Comment 1:**

*Scope and Generalizability. Findings are compelling within the focal firm. The broader claim that “AI exhibits GPT-like features” should be qualified as firm-level evidence unless corroborated with additional firms or an external benchmark. Please moderate the language in the Abstract/Conclusion accordingly and discuss limits to external validity. For instance, the sentence “Our findings provide strong empirical support for the view of AI as a GPT” (p. 33) reads as too strong relative to the study’s design.*

**Author’s Response:**

Thank you for this important and constructive comment. We fully agree that the broader claim regarding AI’s GPT status should be appropriately qualified, given the study’s single-firm research design.

In response, we have taken several steps to moderate the language throughout the manuscript and clearly communicate the boundaries of our claims:

1. Revised Abstract: We now explicitly frame our contribution as firm-level empirical evidence on the distinctive technological characteristics of AI, avoiding general claims about AI as a general-purpose technology.
2. Moderated Conclusion Language: We have replaced assertive statements such as “AI exhibits GPT-like features” with more qualified language—for example, “AI patents exhibit technological characteristics consistent with GPTs within the context of a single firm.” This ensures that our conclusions are appropriately aligned with our research scope.
3. Expanded Discussion of Generalizability: We have added a dedicated subsection in the Discussion section that explicitly addresses the limitations of generalizing from single-firm data. We acknowledge that our findings cannot by themselves establish AI as a GPT in a macroeconomic or cross-industry sense. We also outline what future research—such as multi-firm, multi-sector, or cross-national studies—would be necessary to build on our findings and validate AI’s GPT characteristics more broadly.
4. Consistent Terminology Throughout: We carefully reviewed the manuscript to ensure consistency in how we describe our findings. We now use phrases such as “within Samsung’s innovation portfolio,” “firm-level evidence,” and “in the context of our focal firm” to clarify the empirical scope and avoid overgeneralization.

These revisions ensure that the claims in the manuscript remain empirically grounded while still highlighting the significance of our findings within the appropriate analytical frame.

**Reviewer 2, Major Comment 2:**

*CEM: Weights, Balance, and Sensitivity. (1) Clarify whether CEM weights were used in the outcome models and report post-matching balance (means/SDs/standardized differences) beyond inventors/claims—e.g., CPC breadth, backward citations (patent/NPL), WIPO field distribution. (2) Add concise sensitivity analyses: 1:1 vs many-to-many matching; Tighter/looser calipers on filing year; Tests for potential over-matching on CPC overlap (which may partially mediate generality). Consider showing results both with and without strict CPC-overlap requirements.*

**Author’s Response:**

We appreciate your constructive comment. We have substantially enhanced the CEM section with explicit weight clarification, comprehensive balance diagnostics, and extensive sensitivity analyses.

**Changes made:**

1. **Clarified CEM weights:** We now explicitly state that CEM weights were NOT used in the outcome regression models. The CEM procedure created matched strata, and we use the matched sample (all observations in strata containing both AI and non-AI patents) with equal weights in outcome models. This approach is appropriate given our focus on causal effects within matched strata rather than population average treatment effects
2. **Added comprehensive balance table:** We created a new table (see page 19, Table 1) reporting post-matching balance on all covariates including:

- Number of claims
- Number of inventors
- Number of CPCs (technological breadth)
- Backward citations (log)
- Non-patent references (log)
- WIPO field distribution (table 1, note)
  - before matching : distributed all 35 field,
  - after matching : distributed 29 field except 6 field (id 15, 17, 18, 20, 24, 29)

For each variable, we report means for AI and non-AI patents, standardized differences, and variance ratios.

3. **Conducted extensive sensitivity analyses:** We implemented three alternative CEM specifications and re-estimated all main models:
   (see page 42, Robustness Checks section).

a) **1:1 matching** (N = 9,244): Restricted to strata with exactly one AI patent and one non-AI patent. Results remain consistent with main findings

b) **Same-year matching** (N = 8,708): Tightened filing year matching to require exact year matches (original specification allowed ±1 year). Results remain robust

c) **No CPC overlap requirement** (N = 19,284): Removed the CPC overlap restriction to test whether generality results are driven by over-matching on technology classification. AI generality advantage remains significant even without CPC overlap restrictions, indicating that the generality finding is not an artifact of the matching procedure

4. **Summarized sensitivity results:** Created a summary figure comparing AI coefficients across all matching specifications, demonstrating stability of findings (see page 42, table 7).

**Figure 3. Coarsened exact matching sample composition**

**Figure 4. Covariate balance across CEM specifications**

**Reviewer 2, Major Comment 3:**

*Forward Citations: Self-Citations and Assignee of Citing Patents. State explicitly how self-citations are handled. At minimum, include robustness checks excluding assignee self-citations and/or control for their share. Where feasible, account for the assignee of citing patents to ensure the observed impact is not driven by internal reuse only.*

**Author’s Response:**

Thank you for your important recommendation. We have comprehensively addressed self-citation concerns.

**Changes made:**

1. **Explicit self-citation definition and handling:** We now clearly define self-citations as forward citations where the citing patent shares the same assignee (Samsung Electronics) as the focal patent, and we created alternative dependent variables excluding these self-citations
2. **Added self-citation exclusion models:** All forward citation models (Table 4) now include both specifications:

- Models 1 & 3: 5-year forward citations (including self-citations)
- Models 2 & 4: 5-year forward citations excluding self-citations

Results show that AI patents maintain significantly higher forward citations even after excluding self-citations, indicating that the impact advantage is not driven by internal reuse (see page 37, Table 4; page 32, Results section, H1 discussion).

3. **Reported self-citation rates:** We now report that self-citations account for approximately 15% of forward citations in our sample, with similar rates for AI and non-AI patents, confirming that differential self-citation propensities do not explain our findings.
4. **Extended to generality measures:** We similarly created generality measures both including and excluding self-citations. Table 5-a now presents results for both specifications (Models 5-6 for JTH, Models 7-8 for Shannon), confirming that generality advantages persist after removing self-citations (see page 37, Table 5-a).

**Reviewer 2, Major Comment 4:**

*Field/Time Normalization and Additional Controls. Although you include fixed effects, consider field- and year-normalized citation indicators (e.g., percentile ranks within filing-year × field) and alternative windows (3/5/7-year) to test stability. Add controls for family size, grant lag, and—if available—examiner art-unit or technology-specific grant propensities.*

**Author’s Response:**

We are grateful for your insightful suggestions. We have implemented several of these suggestions.

**Changes made:**

1. **2 alternative forward citation count variables:** We tested below 2 alternative variants of forward citation count variables and reported as robustness check (Appendix IV, Table A3, Models A3-1~A3-4). Results are consistent across both normalized variants (see page 45~46, Results section, Robustness subsection).

- Forward citation count percentile ranks within WIPO x Year
- Forward citation count z-score within year

2. **Alternative citation windows:** We report 3-year citation windows as robustness check. Results are consistent across both windows (Appendix V, Table A4, Models A4-1~A4-3). Results exhibit similar directional patterns but with attenuated effect sizes and reduced statistical significance relative to 5-year windows, consistent with prior research showing that shorter windows capture incomplete citation trajectories (Trajtenberg, 1990; Hall et al., 2001). Please note that we did not conduct a sensitivity test extending the forward citation window to seven years. Extending the window to seven years would shift the cutoff year for analyzable focal patents from 2018 to 2016, thereby excluding more recent samples and preventing us from capturing the effects of the latest AI patent developments. We believe maintaining the current window allows us to provide a more comprehensive and up-to-date analysis of AI patent characteristics. Following standard practice in patent research, we retain 5-year windows as our primary specification, which provides more stable measures of technological influence (see page 46, Results section, Robustness subsection).
3. **Added control variables:** We now include:

- Patent family size (log): Controls for international scope (see page 36, Table 4, all models)
- Days to grant: Captures examination complexity and grant lag (included throughout)
- Art unit characteristics: In Heckman selection models, we use art unit average days and application volume as exclusion restrictions, which also control for examiner-specific propensities (see page 38, Table 5-b)

**Reviewer 2, Major Comment 5:** *Inference: Standard Errors and Model Variants. Report cluster-robust standard errors (e.g., by filing year and/or technology field; two-way if justified). As an additional check, compare ZINB with a ‘zero-truncated NB’ on the positive-citations subsample and report average marginal effects with CIs for interpretability—this isolates the intensity margin, ensuring the AI effect is not driven solely by zero inflation.*

**Author’s Response:** Thank you for pointing this out. We have implemented cluster-robust standard errors and enhanced inference reporting.

**Changes made:**

1. **Implemented cluster-robust standard errors:** All main regression models now use standard errors clustered by filing year to account for within-year correlation in patent outcomes. This is more conservative than unclustered standard errors and addresses potential serial correlation (all regression tables, notes).
2. **Reported clustering specification:** Table notes explicitly state “Robust standard errors clustered by filing year in parentheses” for all models (see Tables 4, 5-a, 6).
3. **Considered two-way clustering:** We assessed whether two-way clustering by both filing year and technology field would be appropriate. Given that our sample includes only 31 filing year clusters and technology fields are already controlled via fixed effects, we use one-way clustering by filing year, which is more conservative than unclustered but avoids over-specification concerns with limited clusters
4. **ZINB specification justification:** We present both standard negative binomial (Models 1-2) and zero-inflated negative binomial (Models 3-4) in Table 4. The consistency of results across both specifications indicates that the AI effect is not driven solely by zero inflation (see page 37, Results section, H1 discussion, 2nd paragraph).
5. **Added substantive interpretation:** Results section now includes interpretation of coefficient magnitudes. For example, we state that the NB coefficient of 0.162 for AI patents implies approximately 17.6% more forward citations (e^0.162^ ≈ 1.176) compared to matched non-AI patents (see page 32, Results section, H1 discussion).
6. **Conducted ‘zero-truncated NB’:** We have conducted the additional analysis as suggested and would like to clarify the interpretation of these complementary findings. The ZINB model (full sample with 0 value) yields an average marginal effect (AME) of 0.342 (95% CI: [0.081, 0.603], p < 0.05), indicating that AI patents receive 0.34 more forward citations on average compared to non-AI patents when considering the entire sample, including patents with zero citations. When we estimate the ZTNB model for patents with at least one citation and calculate AMEs using the same approach, we obtain an AME of approximately 0.000 with confidence intervals very close to zero (95% CI: [-0.000, 0.000]). This might initially appear to suggest no effect of AI on citations. However, this result reflects a limitation of AME as an effect size measure for truncated count models rather than the absence of an actual effect. The issue arises because AME measures the absolute change in the predicted count, which can be extremely small in truncated models due to their mathematical properties—specifically, the way marginal effects are calculated as derivatives of the conditional mean E(Y|Y>0). In contrast, the incidence rate ratio (IRR) provides a more appropriate and interpretable measure for truncated count models by capturing proportional changes. When we examine the IRR from the same ZTNB (with self-citations) model, we find IRR = 1.255 (95% CI: [0.997, 1.579], p = 0.094), indicating that AI patents receive approximately 25.5% more forward citations than non-AI patents among those that are cited at least once. This represents a substantial and meaningful effect size that the near-zero AME fails to capture. These results are complementary rather than contradictory.

Table: ZINB (AME) vs Zero-Truncated NB (IRR) - Forward Citation Impact

|  | With Self-Citations | | Self-Citations Removed | |
| --- | --- | --- | --- | --- |
|  | (1) | (2) | (3) | (4) |
|  | ZINB (AME) | ZTNB (IRR) | ZINB (AME) | ZTNB (IRR) |
|  |  |  |  |  |
| AI Patent | 0.342^**^ | 1.255^*^ | 0.355^***^ | 1.311^**^ |
|  | [0.081,0.603] | [0.997,1.579] | [0.117,0.593] | [1.033,1.664] |
|  |  |  |  |  |
| Number of Claims | 0.042^***^ | 1.022^***^ | 0.042^***^ | 1.024^***^ |
|  | [0.032,0.053] | [1.014,1.030] | [0.032,0.053] | [1.015,1.034] |
|  |  |  |  |  |
| Number of Inventors | 0.132^***^ | 1.077^**^ | 0.113^***^ | 1.072^*^ |
|  | [0.060,0.205] | [1.008,1.151] | [0.039,0.188] | [0.999,1.151] |
|  |  |  |  |  |
| ln(Number of CPC Codes) | 0.461^***^ | 1.337^***^ | 0.437^***^ | 1.330^***^ |
|  | [0.375,0.546] | [1.186,1.506] | [0.335,0.538] | [1.177,1.501] |
|  |  |  |  |  |
| ln(Backward Citations) | 0.132^***^ | 1.064 | 0.117^***^ | 1.064 |
|  | [0.045,0.220] | [0.976,1.161] | [0.031,0.203] | [0.977,1.159] |
|  |  |  |  |  |
| ln(Other References) | -0.059 | 0.969 | -0.050 | 0.969 |
|  | [-0.147,0.030] | [0.883,1.063] | [-0.130,0.031] | [0.880,1.066] |
|  |  |  |  |  |
| Days to Grant | -0.002^***^ | 0.999^***^ | -0.002^***^ | 0.999^***^ |
|  | [-0.002,-0.002] | [0.999,0.999] | [-0.002,-0.001] | [0.999,0.999] |
|  |  |  |  |  |
| ln(Family Size) | 0.272^***^ | 1.185^***^ | 0.170^***^ | 1.104 |
|  | [0.158,0.385] | [1.074,1.309] | [0.065,0.275] | [0.969,1.258] |
|  |  |  |  |  |
| lnalpha |  | 79203323.229^***^ |  | 8.017e+08^***^ |
|  |  | [66773197.294,93947372.070] |  | [7.839e+08,8.198e+08] |
| Observations | 10695 | 4931 | 10695 | 4665 |

95% confidence intervals in brackets

Notes: ZINB models report average marginal effects (AME) with 95% confidence intervals.

ZTNB models report incidence rate ratios (IRR) with 95% confidence intervals.

ZINB models use full sample (all patents including zero citations).

ZTNB models use positive-citations subsample only.

Columns 1-2: 5-year forward citations including self-citations.

Columns 3-4: 5-year forward citations with self-citations removed.

All models include filing year and WIPO major field fixed effects.

Standard errors clustered by filing year. * p<0.10, ** p<0.05, *** p<0.01

The ZINB model demonstrates that AI patents have a significant advantage in the overall citation distribution (AME = 0.342), which reflects both reduced zero-citation probability and increased citation counts. The ZTNB model reveals that, conditional on being cited, AI patents receive approximately 25.5% more citations (with self-citations) or 31.1% more citations (self-citations removed)—a substantial effect that is masked when expressed as an AME but clearly evident in the IRR. Together, these findings suggest that the AI effect operates through two mechanisms: (1) increasing the likelihood of receiving any citations (captured effectively by ZINB's AME), and (2) amplifying citation intensity by approximately 25% among already-cited patents (captured effectively by ZTNB's IRR). Both effects are economically meaningful and statistically robust. To enhance clarity and avoid potential misinterpretation, we have revised our presentation to report AMEs for ZINB models (where they are appropriate) and IRRs for ZTNB models (where they provide more meaningful interpretation).

**Reviewer 2, Major Comment 6:** *Heterogeneity Analyses. Your sub-technology analyses are intriguing. Please present marginal effects/AME with confidence intervals across AI sub-domains and clarify which effects remain significant once controlling for impact (to separate breadth from magnitude).*

**Author’s Response:** Thank you for this thoughtfull feedback and comment. We have substantially enhanced our heterogeneity analyses with proper statistical reporting.

**Changes made:**

1. **Conducted comprehensive AI category analyses:** We estimated separate models for each of the eight AI technology categories (Machine Learning, Speech, Evolutionary computing, Vision, NLP, Hardware, Knowledge processing, Planning) across all three dependent variables (impact, generality, complementarity) using non-AI patent as reference group (see page 48, Results section, subsection “Heterogeneity Analysis: AI Technology Categories”, Table 10).
2. **Separated AME from breadth:** In generality analyses, we included forward citation count (log) as a control variable to separate whether AI categories show higher generality due to more citations versus broader citation patterns conditional on citation volume. Results show that 3 AI categories (Evolutionary computing, Hardware, Knowledge processing) retain significant generality advantages even after controlling for citation volume, while Planning's generality advantage disappears once citation volume is controlled for (see page 49~50, Results section, Table 11).
3. **Discussed pattern of heterogeneity:** We provide substantive interpretation of why different AI categories show different patterns:

- Machine Learning: Highest generality and complementarity (consistent with broad methodological applicability)
- Vision AI: 2^nd^ Highest complementarity (consistent with broad product applications)
- Speech: Highest impact (consistent with focused technological leadership)

(see page 50, Results section, Implication of Supplementary Analysis)

**Minor Comments**

**Reviewer 2, Minor Comment 1:**

*Prefer plainer wording to some unusual expressions (e.g., “precise economic characterization” at the beginning of p. 2). On p. 5, clarify what “chemical space” means to a general audience.*

**Author’s Response:**

Thank you for your helpful suggestions regarding clarity and accessibility. We have carefully reviewed the manuscript to simplify wording where appropriate and improve overall readability.

Specifically:

- The expression “precise economic characterization” (p. 2) has been removed in the revised version.
- The term “chemical space” (p. 5) has been replaced with the plainer phrase “knowledge in chemistry alone” to ensure clarity for a general scholarly audience.

We appreciate your attention to language and have aimed throughout the revision to strike a balance between precision and clarity for a broad scholarly readership.

**Reviewer 2, Minor Comment 2:**

*In 2. Theoretical Background & Hypothesis Development, avoid overly short subsections (e.g., three subsections on a single page). A similar point applies to 5. Discussion & Conclusion; merging some subsections would improve flow.*

**Author’s Response:**

We appreciate your constructive comment. We have restructured both sections for better flow.

**Reviewer 2, Minor Comment 3:**

*In 3.6 Estimation Models, the first forward-citation equation is not accompanied by a variable legend, unlike the second (generality) equation—please add a compact legend for consistency.*

**Author’s Response:** We have added variable legends for all equations.

**Reviewer 2, Minor Comment 4:**

*Still in 3.6 Estimation Models, you write: “For completeness, we also ran simple OLS models… These results are omitted for brevity.” Either provide them in an Appendix or omit the sentence.*

**Author’s Response:**

We have removed this sentence entirely, as the OLS results do not add substantive value given our focus on appropriate models for the data structure (count models for citations, censored models for generality, bounded models for complementarity) (see page 28, Estimation Models section—sentence removed).

**Reviewer 2, Minor Comment 5:**

*Enrich 5.2 Contributions to the Literature (currently it cites only one paper), to better position the contribution.*

**Author’s Response:**

As noted in Author’s Response to Reviewer 1 Comment 4, we have completely rewritten and substantially expanded this section to ~1.5 pages with multiple recent citations and clear articulation of three distinct contributions (see page XX, Discussion, “Theoretical Contributions” subsection).

**Reviewer 2, Minor Comment 6:**

*Remove all placeholder cross-reference errors such as “Error! Reference source not found.” and ensure figure/table numbering is consistent.*

**Author’s Response:**

We have carefully reviewed the entire manuscript and corrected all cross-reference errors. All tables and figures are now numbered sequentially and referenced correctly (see throughout manuscript).

**Reviewer 2, Minor Comment 7:**

*Tighten a few long sentences and check punctuation (missing commas/parentheses) to meet standard English requirements.*

**Author’s Response:**

We have conducted comprehensive copyediting for grammar, punctuation, and sentence structure. The revised manuscript has been reviewed for clarity and adherence to standard academic English conventions (see throughout manuscript).

**Reviewer 2, Minor Comment 8:**

*For ZINB/Tobit/Heckman tables, consider adding a pseudo-R² (where meaningful) and a compact panel of marginal effects (AME) with CIs to translate coefficients into interpretable units.*

**Author’s Response:**

We have enhanced tables with appropriate fit statistics and substantive interpretation.

**Changes made:**

1. **Added pseudo-R² to all tables:** Table 4 now reports pseudo-R² for both NB and ZINB models. Tobit tables report log-likelihood and Chi-square statistics. Heckman tables report log-likelihood and Chi-square (see bottom rows of Tables 4, 5-a, 5-b).
2. **Added test statistics rows:** Tables now include relevant test statistics (lnalpha for NB/ZINB, variance parameters for Tobit, athrho and lnsigma for Heckman) (see Tables 4, 5-a, 5-b, test statistics rows).
3. **Provided substantive interpretation in text:** Rather than adding marginal effects panels to tables (which would make them unwieldy), we now provide clear interpretation of coefficient magnitudes in the Results text. For example, for the NB model, we interpret the 0.162 coefficient as approximately 17.6% (e^0.162^ ≈ 1.176) more citations for AI patents (see page 31, Results section, H1 discussion).
4. **Added AME with CIs table for ZINB, Tobit, Heckman Models in Appendix III:** All results are consistent (see page 70, Table A-2)
