# Supplementary material for: Technological impact, generality, and complementarity of artificial intelligence patents: Evidence from Samsung Electronics
Source: PLoS One. 2026 May 5;21(5):e0346056. doi: 10.1371/journal.pone.0346056 (PMC13143100; doi:10.1371/journal.pone.0346056)
Supplement: Attachment — Submitted filename: Response 20.12.2025 PONE.docx [file pone.0346056.s005.docx]

Response 20.12.2025

As it was added before the paper entitled „Artificial Intelligence as a General-Purpose Technology? Insights from Samsung’s Patents” has an interesting topic based on using AI and modelling. But in order to be proposed for publication, the authors need to make the following improvements:

2.The **THEORETICAL BACKDROP & HYPOTHESIS DEVELOPMENT** is suffering of adding new and updated sources. As the authors use a topic and analyse it under the impact of AI, the authors need to use upadated references. Only one source per described section it does not indicating the improvement according to the rquests.

The authors must ad t least 2-3 sources especially from 2025, duet o the dynamism of the digital impact.

4.**Contribution to Literature** must be improved. The authors used only one source (from 1995), so to measure the gap between the study and the literature in the filed, they have to use a few new and updated sources.

The authors must ad a few updated sources from 2025.

5.**Practical implications** need also a more carefull description. It is indicated to describe PI on categories (write them using italic writing for sub-titles): for employees, for managers, for the industry, for society.

The authors added just implications for firms and policymakers, the other requested implications are still missing. More careful attention, please!

6.**References** must be updated, as the requirement, being a dynamic field under the impact of AI.

The authors must add a few more sources especially from 2025!!!

Having these proposals in view, the paper receives major revision, so please pay attention to the indicated improvement measures!
