# Supplementary material for: Technological impact, generality, and complementarity of artificial intelligence patents: Evidence from Samsung Electronics
Source: PLoS One. 2026 May 5;21(5):e0346056. doi: 10.1371/journal.pone.0346056 (PMC13143100; doi:10.1371/journal.pone.0346056)
Supplement: Attachment — Submitted filename: Response PONE-D-25-42932R1.docx [file pone.0346056.s006.docx]

**Response to Reviewers**

**Manuscript ID:** PONE-D-25-42932R1

**Title:** Technological Impact, Generality, and Complementarity of Artificial Intelligence Patents: Evidence from Samsung Electronics

We sincerely thank the Academic Editor and both Reviewers for their constructive feedback on our revised manuscript. We have carefully addressed all comments, and we believe the manuscript has been substantially strengthened as a result. Below, we provide point-by-point responses to each comment. All page numbers refer to the revised manuscript with track changes.

**Response to Academic Editor**

**Editor’s Comment:** “While the manuscript has considerably improved, the Reviewers – and particularly Reviewer 1 – raise important concerns regarding the need for further engagement with the literature. I support Reviewer 1’s view and ask you to address these concerns very thoroughly, but also to address the point Reviewer 2 has made.”

**Response:** We appreciate the Editor’s guidance. We have thoroughly addressed both Reviewers’ concerns by (1) adding recent 2025 publications throughout the manuscript, (2) restructuring the Practical Implications section with detailed stakeholder categories, (3) ensuring consistent GPT framing throughout, (4) softening language around complementarity interpretation, and (5) adding a dedicated paragraph explaining what drives generality differences. Details are provided below.

**Response to Reviewer #1**

**Comment 1: Theoretical Backdrop needs updated sources**

**Reviewer’s Comment:** The THEORETICAL BACKDROP & HYPOTHESIS DEVELOPMENT is suffering of adding new and updated sources. As the authors use a topic and analyse it under the impact of AI, the authors need to use updated references. Only one source per described section it does not indicating the improvement according to the requests. The authors must add at least 2-3 sources especially from 2025, due to the dynamism of the digital impact.

**Response:** We appreciate the reviewer’s important suggestion regarding the need to incorporate more recent and up-to-date literature, particularly given the rapid evolution of AI technologies and their economic and technological impacts. In response, we have substantially revised Section 2 (Theoretical Backdrop & Hypothesis Development) to better reflect the current state of the literature.

Specifically, we have incorporated more than ten recent references, including multiple studies published in 2024–2025, that explicitly examine AI through the lens of general purpose technologies (GPTs), technological pervasiveness, and complementarity. These additions include recent conceptual and empirical contributions that use patent-based indicators, firm-level outcomes, and supply-chain evidence to assess AI’s GPT-like characteristics. As a result, each conceptual subsection in Section 2 is now supported by multiple, up-to-date sources, rather than relying on a single reference.

In addition, we restructured the opening paragraphs of Section 2 to foreground these recent studies and to more clearly position our hypotheses within the evolving GPT and AI innovation literature. This revision strengthens the theoretical grounding of the paper and directly addresses the reviewer’s concern about reflecting the dynamism of digital and AI-driven technological change.

We believe these revisions substantially improve the timeliness, depth, and relevance of the theoretical framework.

*“Given AI’s expanding applicability and innovation-enabling potential, a growing body of scholarship has argued that AI constitutes a contemporary GPT, comparable to historical examples such as electricity, the steam engine, and computing technologies (Brynjolfsson et al., 2021; Cockburn et al., 2019; Czarnitzki et al., 2023; Rammer et al., 2022). More recent empirical studies have begun to systematically assess AI’s GPT characteristics using patent-based indicators. For example, Hötte et al. (2025) compare multiple patent-based definitions of AI and find that AI consistently exhibits high rates of growth, technological generality, and complementarity across specifications—key hallmarks of GPTs. Similarly, Dong et al. (2025) show that firms specializing in GPT-related R&D experience greater cross-industry expansion in supply chains, with these effects moderated by the breadth and depth of firms’ technology portfolios.*

*Taken together, these theoretical and empirical insights provide a strong foundation for viewing AI as a transformative and broadly applicable technology, or GPT. The GPT perspective provides a useful lens for conceptualizing AI as a foundational technology that catalyzes innovation across diverse technological domains (Lin & Maruping, 2025). Agrawal et al. (2019) emphasize AI’s capacity to reduce the cost of prediction, thereby enabling new decision-making processes, organizational practices, and business models. Similarly, Furman and Seamans (2019) highlight AI’s productivity-enhancing effects in sectors such as transportation, healthcare, and education. Together, these studies suggest that AI can contribute to long-run economic growth by increasing total factor productivity and stimulating innovation across a broad set of industries.*

*To examine how AI innovations may exhibit distinctive technological characteristics, we draw on the defining features of GPTs: continuous technological improvement, pervasiveness across a wide range of uses, and innovational complementarities with existing and future technologies (Bresnahan & Trajtenberg, 1995; Lipsey et al., 1998). Given the well-documented rapid technological advancement of AI, this study focuses on the latter two dimensions—pervasiveness and complementarity. In the following sections, we develop hypotheses related to each dimension.”*

**References added:**

- Dong, F., Zhang, G., & Mei, L. (2025). General purpose technology R&D specialization and supply chain cross-industry expansion: The moderation effect of technology portfolio breadth and depth. *Technology in Society*, 82, 102889. <https://doi.org/10.1016/j.techsoc.2025.102889>
- Hötte, K., Tarannum, T., Verendel, V., & Vezzani, A. (2025). Measuring artificial intelligence: A systematic assessment and implications for governance. *Industrial and Corporate Change*. Advance online publication. https://doi.org/10.1093/icc/dtaf047
- Lin, Y. K., & Maruping, L. M. (2025). Organizing for AI Innovation: Insights From an Empirical Exploration of US Patents. MIS Quarterly, 49(3), 1095-1122.

**Comment 2: Contribution to Literature needs updated sources**

**Reviewer’s Comment:** Contribution to Literature must be improved. The authors used only one source (from 1995), so to measure the gap between the study and the literature in the field, they have to use a few new and updated sources. The authors must add a few updated sources from 2025.

**Response:** We thank the reviewer for this valuable suggestion regarding the need to strengthen and update the paper’s contribution to the literature. In response, we have carefully revised Section 5 to better situate our study within the rapidly evolving literature on AI and general purpose technologies.

Specifically, we have substantially revised Section 5.1 to more clearly contextualize our research questions and findings within recent theoretical and empirical examinations of AI as a GPT, drawing on newly published studies that emphasize technological pervasiveness, complementarity, and firm-level impacts. More importantly, we have significantly expanded the Contribution to Literature section (Section 5.2) by adding five recent firm-level AI studies published in 2025. These additions allow us to explicitly position our contribution relative to the current frontier of AI–GPT research, rather than relying primarily on foundational GPT work from the 1990s.

As a result, the revised contribution section now clarifies how our patent-level analysis complements and extends recent firm-level and supply-chain–level studies, and how our findings on impact, generality, and complementarity offer novel empirical insights into the GPT-like nature of AI. We believe these revisions substantially strengthen the paper’s contribution and directly address the reviewer’s concern regarding the use of updated and relevant literature.

The revised passage now reads:

Section 5.1

*“While AI technologies have attracted substantial attention from business managers and technologists, a systematic understanding of their nature and broader technological impacts remains essential for designing effective strategies and policies (Capraro et al., 2024). Despite this importance, empirical evidence on the technological characteristics and systemic implications of AI remains limited and fragmented (Hötte et al., 2025; Lin & Maruping, 2025; Petralia, 2020). In this study, we address this gap by examining the “GPTness” of AI patents, using Samsung Electronics as a representative case.*

*…*

”

Section 5.2

*“…*

*While recent studies have begun to validate these characteristics at the macro level—through analyses of citation diversity and co-classification patterns (Hötte et al., 2022, 2025; Lee et al., 2025)—firm-level evidence remains scarce. Notably, recent firm-level AI research has primarily focused on AI’s impact on corporate outcomes, such as environmental performance (Liu et al., 2025), ESG ratings (Xie et al., 2025), and innovation performance (Ma et al., 2025), treating AI as an input variable rather than examining the structural characteristics of AI technology itself. Recent patent-based research has examined organizational antecedents of AI innovation, such as how firm structure and cross-functional collaboration shape AI patenting outcomes (Lin & Maruping, 2025). However, these studies focus on what drives AI patent generation rather than on the intrinsic technological characteristics of AI patents themselves. Similarly, Yavuz and Çalik (2025) analyzed lagged effects of AI/ML patent intensity on firm performance, treating AI patents as inputs to financial outcomes. Our study complements this stream by examining the technological structure of AI patents—specifically their impact, generality, and complementarity—rather than their downstream performance effects.”*

**References added:**

- Capraro, V., Lentsch, A., Acemoglu, D., Akgun, S., Akhmedova, A., Bilancini, E., Bonnefon, J.-F., Brañas-Garza, P., Butera, L., Douglas, K. M., Everett, J. A. C., Gigerenzer, G., Greenhow, C., Hashimoto, D. A., Holt-Lunstad, J., Jetten, J., Johnson, S., Kunz, W. H., Longoni, C.,…Viale, R. (2024). The impact of generative artificial intelligence on socioeconomic inequalities and policy making. PNAS Nexus, 3(6), pgae191. https://doi.org/10.1093/pnasnexus/pgae191
- Dong, F., Zhang, G., & Mei, L. (2025). General purpose technology R&D specialization and supply chain cross-industry expansion: The moderation effect of technology portfolio breadth and depth. *Technology in Society*, 82, 102889. <https://doi.org/10.1016/j.techsoc.2025.102889>
- Hötte, K., Tarannum, T., Verendel, V., & Vezzani, A. (2025). Measuring artificial intelligence: A systematic assessment and implications for governance. *Industrial and Corporate Change*. Advance online publication. https://doi.org/10.1093/icc/dtaf047
- Lin, Y. K., & Maruping, L. M. (2025). Organizing for AI Innovation: Insights From an Empirical Exploration of US Patents. MIS Quarterly, 49(3), 1095-1122.
- Liu, Y., Wang, B., & Song, Z. (2025). Promoting or inhibiting: The impact of artificial intelligence application on corporate environmental performance. International Review of Financial Analysis, 97, 104123. https://doi.org/10.1016/j.irfa.2024.104123
- Ma, Y., Zhang, W., Ma, C., Ai, Y., & Hu, J. (2025). Artificial intelligence, data elements, digital economy, and corporate innovation performance. International Review of Economics & Finance, 99, 103–120. https://doi.org/10.1016/j.iref.2025.103248
- Xie, H., Luo, J., & Tan, X. (2025). Artificial intelligence technology application and corporate ESG performance—Evidence from national pilot zones for artificial intelligence innovation and application. Frontiers in Artificial Intelligence, 8, 1643684. https://doi.org/10.3389/frai.2025.1643684
- Yavuz, M. S., & Çalik, H. (2025). AI and ML patent intensity and firm performance: A machine learning-based lagged analysis. European Research on Management and Business Economics, 31(3), 100291.

**Comment 3: Practical Implications needs category-specific descriptions**

**Reviewer’s Comment:** Practical implications need also a more careful description. It is indicated to describe PI on categories (write them using italic writing for sub-titles): for employees, for managers, for the industry, for society. The authors added just implications for firms and policymakers, the other requested implications are still missing. More careful attention, please!

**Response:** Thanks for your comments. We have now completely restructured Section 5.3 with four distinct stakeholder categories using italic subtitles as requested:

*For managers* – We discuss the strategic value of internal AI expertise, portfolio diversification across AI categories, and AI as a systematic enabler to be integrated across the innovation portfolio rather than siloed within specialized units.

*For employees* – We address the durable career value of AI-related skills, the transferability of AI competencies across functions and sectors, and the importance of combining AI expertise with domain-specific knowledge.

*For industry* – We explain how AI innovations sustain relevance through cross-domain applications, offering stronger incentives for sustained AI investment even under conditions of creative destruction.

*For society and policymakers* – We discuss AI’s role as transformative infrastructure justifying policy support, the heterogeneity across AI domains requiring differentiated policy approaches, and international competitiveness implications.

The complete revised text spans approximately two pages and provides substantive implications for each stakeholder group.

We also incorporated a recent publication (Francis Xavier et al., 2025) examining AI’s impact on job autonomy and creative self-efficacy, which directly supports our discussion of implications for employees.

Francis Xavier, D., Korunka, C., & Reiter-Palmon, R. (2025). AI integration and workforce development: Exploring job autonomy and creative self-efficacy in a global context. PLOS ONE, 20(6), e0319556. https://doi.org/10.1371/journal.pone.0319556

**Response to Reviewer #2**

We thank Reviewer #2 for the positive assessment and thoughtful suggestions for final refinements. We have addressed all three points raised.

**Comment 1: GPT framing consistency**

**Reviewer’s Comment:** While the new version moderates the language compared with the prior version, the manuscript should remain fully consistent throughout (abstract, discussion, conclusion) in presenting the findings as evidence consistent with GPT-like characteristics within the focal firm, rather than as a broad characterization of AI as a GPT in general. For example, in the 1.Introduction the text states that AI’s properties are ‘defining features of GPTs.’ Suggestion: Rephrase such statements to make clear that these are GPT-like features tested in this study within a focal firm context.

**Response:** We appreciate this guidance and have revised the manuscript throughout to ensure consistent, appropriately qualified framing. Key changes include:

**Introduction (p. 2):**

- Changed: “all of which are defining features of general-purpose technologies (GPTs)”
- To: “all of which are often discussed as characteristics associated with general-purpose technologies (GPTs). This study empirically examines whether these GPT-like features can be observed in AI patents within a single firm context.”

**Introduction (p. 2):**

- Changed: “we examine three defining characteristics of GPTs”
- To: “we examine three characteristics commonly associated with GPTs”

**Discussion (p. 46):**

- Changed: “characteristics consistent with general purpose technologies”
- To: “characteristics consistent with those commonly attributed to general-purpose technologies”

We have also reviewed the Abstract, Discussion, and Conclusion sections to ensure the language consistently presents our findings as firm-level evidence of GPT-like characteristics rather than definitive claims about AI as a GPT in general.

**Comment 2: Understanding complementarity**

**Reviewer’s Comment:** The new measure is interesting and well motivated, but some statements interpret the results in terms of ‘purposeful/deliberate’ technological coupling. Given the observational nature of the study, I encourage the authors to soften the intentional language and present the results as systematic patterns consistent with complementarity, avoiding claims about intent that the data cannot directly observe. Suggestion: Replace ‘purposeful/intentional/deliberate’ with ‘systematic / non-random / consistent with complementarity’.

**Response:** We agree that our observational data cannot directly reveal managerial intent, and we have revised all relevant passages accordingly:

**Hypothesis Development (p. 8):**

- Changed: “purposeful integration” → “systematic integration”
- Changed: “reflects such strategic alignment rather than opportunistic or random recombination” → “exhibits patterns consistent with strategic alignment rather than random recombination”

**Methodology (p. 18):**

- Removed the phrase “indicating purposeful synergistic relationships rather than coincidental overlap” and revised the description of the complementarity measure using more cautious language to avoid overinterpretation.

**Results (p. 31):**

- Changed: “AI innovations systematically integrate with diverse technologies more frequently than random expectations would predict, indicating purposeful synergistic relationships rather than coincidental overlap”
- To: “AI innovations co-occur with diverse technologies more frequently than random expectations would predict, exhibiting systematic, non-random patterns consistent with complementarity rather than coincidental overlap”

**Discussion (p. 48):**

- Changed: “intentional, rather than incidental, integration”
- To: “systematic, rather than incidental, co-occurrence patterns”

**Comment 3: Connecting results to what drives generality**

**Reviewer’s Comment:** The paper would benefit from a clearer explanation of what is driving differences in the generality indices... Readers may still ask: is higher generality mainly because AI patents are more cited, or because they are genuinely broader even conditional on citations? Suggestion: Add a short paragraph in the 5. Discussion and conclusion section that explicitly states (in plain terms) what your results imply.

**Response:** We thank the Reviewer for this excellent suggestion. We have added a dedicated paragraph at the end of Section 5.1 (Summary of Findings, **p. 47**) that directly addresses this question:

“One potential concern is what underlies the observed differences in generality between AI and non-AI patents—specifically, whether the higher generality of AI patents reflects genuine technological breadth or merely results from their receiving more citations. We argue that the increased generality of AI patents is driven by their inherent cross-domain applicability, rather than by citation volume alone.

First, the coefficient on the AI indicator remains positive and statistically significant in the Tobit models even after controlling for the number of forward citations (Models 5–8 in Table 5-a). This indicates that AI patents exhibit broader technological influence conditional on citation intensity. Second, the Heckman selection models (Table 5-b), which explicitly separate the likelihood of receiving a sufficient number of citations (selection stage) from generality conditional on citations (outcome stage), show positive effects of AI patents in both stages. This finding suggests that the generality advantage of AI patents is not driven solely by selection into the cited sample. Third, supplementary analyses comparing total generality (without citation controls) and pure generality (controlling for forward citations) in Table 11 demonstrate that AI subfields such as vision, machine learning, and speech retain levels of generality comparable to the baseline specifications, even after accounting for citation intensity.

Taken together, these results indicate that the higher generality scores observed for AI patents reflect substantive technological breadth across multiple domains, rather than merely increased citation accumulation.”
